# Hepatoprotective effects of ginsenoside Rb_1_ from *Panax ginseng* on early liver injury via DNA repair activation and nuclear envelope stabilization

**DOI:** 10.1016/j.chmed.2026.05.001

**Published:** 2026-05-06

**Authors:** Fei Gao, Haiyun Gao, Yun Lin, Longyan Chen, Zheng Qi, Miao Yu

**Affiliations:** a Engineering Research Center of Natural Antineoplastic Drugs, Ministry of Education, Harbin University of Commerce, Harbin 150076, China; b Department of Oncology, Shanghai East Hospital, School of Medicine, Tongji University, Shanghai 200120, China

*To the Editor*,

With growing emphasis on early disease prevention within the One Health framework, the exploration of traditional medicinal resources with organ-protective functions is of considerable significance (Li, 2024). Ginsenoside Rb_1_ (GRb_1_), one of the major active constituents in *Panax ginseng* C. A. Mey., is well known for its antioxidant and hepatoprotective properties (Song, Chen, & Li, 2024). Acetamiprid (ACE) was chosen as the modeling agent in this study due to its widespread use and ubiquitous environmental presence (Zhang et al., 2022, Deng et al., 2022), which made chronic exposure a realistic and relevant concern. The liver, as a vital organ responsible for metabolism and detoxification, is particularly susceptible to injury. Identifying safe and effective strategies to mitigate hepatotoxicity has become a key focus in the field of public health. Our results firstly demonstrate that GRb_1_ markedly attenuates early liver injury by enhancing DNA repair efficiency through activation of the non-homologous end joining (NHEJ) pathway and by stabilizing the nuclear envelope. Comprehensive analyzes including histopathology, serum biochemical assays, transcriptomic profiling, observation of nuclear morphology, and protein expression studies reveal that GRb_1_ preserves hepatocyte genomic and structural integrity by upregulating nuclear envelope-associated proteins [nuclear envelope spectrin repeat protein 1 (Nesprin1) and Sad1 and UNC84 domain containing 1/2 (SUN1/2)] and modulating Lamin A/C expression. These findings provide novel mechanistic evidence for the applications of medicinal and edible herbal resources in the prevention of organ dysfunction, and underscore the potential of *P. ginseng*-derived constituents as early interventions within the framework of preventive medicine.

## 1 GRb_1_ alleviates liver injury and attenuates pathological liver changes

To investigate the hepatoprotective effect of GRb_1_, male C57BL/6J mice (Jiao et al., 2025) were exposed to ACE (5 mg/kg) to establish an early liver injury model (Fig. S1A), and detailed dosing rationale is provided in the supplementary materials. GRb_1_ was administered at GRb_1_ low-dose (GRb_1_-L, 12.5 mg/kg), medium-dose (GRb_1_-M, 25 mg/kg), high-dose (GRb_1_-H, 50 mg/kg), and glutathione (GSH, 50 mg/kg) as a positive control (Zhang et al., 2023). Mice in control group were given intragastric administration of equal amounts of sterile normal saline.

Histopathological evaluation of hematoxylin and eosin (HE) staining revealed pronounced hepatic injury in the model group, characterized by hepatocellular edema (highlighted in green boxes), ballooning degeneration, and disorganized hepatic architecture (Fig. S1B). GRb_1_-H markedly alleviated these pathological changes, as evidenced by reduced vacuolar degeneration and improved hepatic architecture. In the GRb_1_-M group, hepatocytes continued to exhibit translucent degeneration. Nuclear positioning was more centralized, and cellular organization was more compact than in the model group. In the GRb_1_-L group, no significant improvement in liver injury was observed. Vacuolated regions remained extensive (highlighted in red boxes), and hepatocyte alignment showed limited restoration, which reflected insufficient protective efficacy at this dose. Compared with the model group, hepatoprotective effects of GRb_1_ led to a dose-dependent decrease in liver index (Fig. S1C).

Consistently, GRb_1_ administration was accompanied by improvement in serum biochemical parameters related to liver function and cellular stress. Levels of alanine aminotransferase (ALT) and aspartate aminotransferase (AST) were reduced following GRb_1_ intervention (Fig. S1D). In parallel, enhanced antioxidant capacity was associated with increased activities of superoxide dismutase (SOD) and catalase (CAT), as well as reduced malondialdehyde (MDA) levels (Fig. S1E). Notably, hepatic levels of 8-hydroxy-2′-deoxyguanosine (8-OHdG) (Fig. S1F), a marker of DNA damage, were decreased in GRb_1_-treated groups, accompanied by reduced oxidative DNA damage under early injury conditions.

To elucidate the molecular mechanisms by which GRb_1_ alleviates hepatotoxicity, transcriptomic analysis was performed on liver tissues from each group. Kyoto encyclopedia of genes and genomes (KEGG) pathway enrichment analysis revealed that GRb_1_ significantly regulated multiple biological processes (BPs) closely related to hepatic metabolism. Given the liver’s central role in detoxification, enrichment of these metabolic pathways represented a characteristic transcriptional response under toxicant exposure conditions. Beyond metabolic pathways, GRb_1_ was also found to significantly activate signaling pathways related to DNA damage repair, particularly the NHEJ pathway (Fig. S1G). This suggested that GRb_1_ may alleviate oxidative damage by enhancing the repair of DNA double-strand breaks (DSBs). GRb_1_ treatment led to a dose-dependent recovery in the expression of key NHEJ-related genes (Fig. S1H, I), which may contribute to the maintenance of genomic stability under early hepatic injury conditions.

## 2 GRb_1_ promotes morphological restoration of hepatocyte membrane and nucleus

GRb_1_ is a highly glycosylated triterpenoid saponin with low oral bioavailability due to its high polarity and molecular weight. Nevertheless, it undergoes extensive biotransformation by intestinal microbiota and hepatic metabolism, giving rise to bioactive metabolites that contribute to its reported hepatoprotective and antioxidant effects. These characteristics support its use in studies focusing on metabolism-dependent and preventive mechanisms of liver injury. GRb_1_ exhibited no cytotoxicity across a concentration range of 0–300 μmol/L (Fig. S2A). The most pronounced enhancement in cell viability was observed at 100 μmol/L. Based on this concentration, serial doses of 50, 25, and 12.5 μmol/L, corresponding to 1/2, 1/4, and 1/8 of 100 μmol/L, were designated as the high, medium, and low doses, respectively (Fig. S2B). Subsequently, Hoechst 33,342 and 3,3′-dioctadecyloxacarbocyanine perchlorate (DiO) fluorescent dyes were used to stain the nucleus (blue) and cell membrane (green), respectively. Under ACE exposure (4.5 mmol/L), hepatocyte membranes exhibited distinct protrusions, and nuclear envelope boundaries became increasingly blurred, indicating early structural damage to both cellular and nuclear membranes. GRb_1_ progressively alleviated structural damage to both the cellular and nuclear membranes. These structural improvements were considered to provide favorable spatial and structural conditions for subsequent DNA damage repair processes, thereby contributing to the maintenance of genomic stability (Fig. S2C).

To further evaluate the protective effect of GRb_1_ on nuclear envelope damage, the expression levels of four key nuclear envelope-associated proteins Nesprin1, SUN1, SUN2, and Lamin A/C were examined (Fig. S2D). GRb_1_ partially restored the normal expression of nuclear envelope structural proteins and alleviated damage to the nuclear envelope. The expression levels of key nuclear envelope components, including Nesprin1, SUN1, and SUN2, were markedly increased following GRb_1_ treatment. These effects contributed to the restoration of nuclear architecture and improved nuclear mechanical stability. As a result, the nucleus exhibited enhanced resistance to external mechanical stress. Together, these findings demonstrate the integrated protective actions of GRb_1_, encompassing cytoprotective, antioxidant, and anti-stress properties. Notably, although the nuclear envelope structure was largely repaired, the expression of Lamin A/C remained elevated relative to the control group. This phenomenon referred to as defensive compensation is common in the early stages of chronic damage repair and suggests that hepatocytes remained in a stress-responsive state following oxidative and genotoxic insults (Liu et al., 2025). The upregulation of Lamin A/C is considered an adaptive response to nuclear instability. In the short term, this response may help preserve nuclear structural integrity (Fig. S2E and S3). However, sustained elevation of Lamin A/C expression may also reflect incomplete recovery of hepatic tissue and an ongoing risk of cellular damage.

**Fig. 1:**
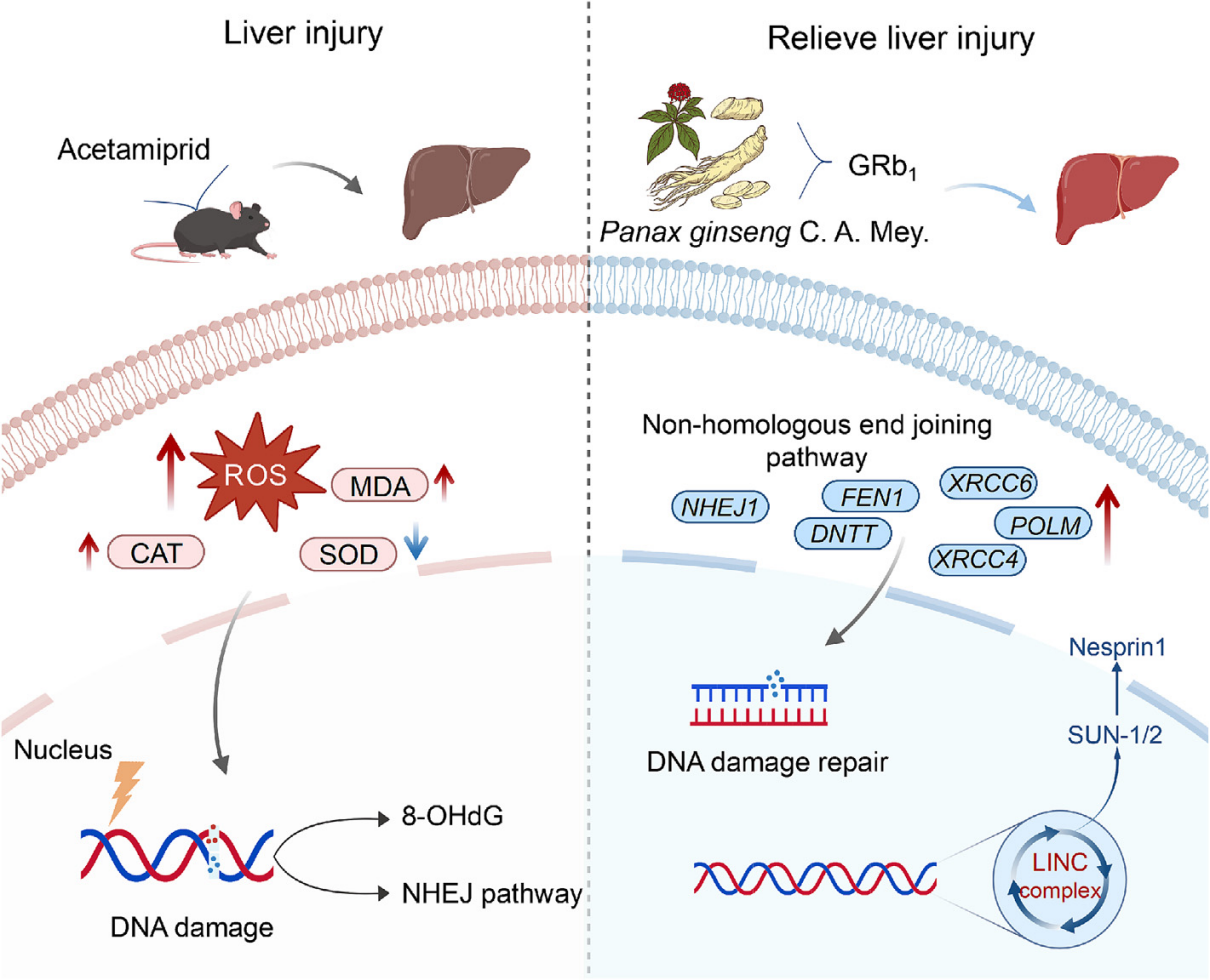
GRb_1_ alleviates early liver injury induced by acetamiprid via promoting DNA repair and stabilizing the nuclear envelope.

Overall, GRb_1_ alleviates acetamiprid-induced disruption of nuclear envelope organization through regulation of nuclear envelope associated proteins, thereby improving nuclear structural stability under stress conditions. Maintenance of nuclear integrity provides a more stable intranuclear environment and supports the preservation of genomic homeostasis during cellular injury. Importantly, this structural perspective complements the observed activation of DNA damage response and repair pathways, as efficient DNA repair depends on an intact nuclear architecture for proper damage sensing and repair factor coordination. Thus, the analysis of nuclear envelope integrity extends the functional findings on DNA repair by addressing the structural conditions that enable effective genome maintenance. Collectively, these findings highlight a previously underappreciated role of GRb_1_ in maintaining nuclear integrity under toxic stress and underscore its potential as a protective agent in the prevention of liver injury (Fig. 1).

Notwithstanding this, several limitations should be acknowledged. Direct functional assays of nuclear envelope integrity (e.g., nucleoporin immunofluorescence or dye exclusion assays) and DSB-specific measurements (such as *γ*H2AX staining or comet assays) are not included. In addition, upstream signaling pathways linking GRb_1_ to antioxidant defense and DNA repair, as well as functional validation of metabolically enriched pathways, are not systematically examined. Histological evaluation relies on representative HE staining without blinded semi-quantitative scoring, and molecular docking analyses are not performed. These limitations will be addressed in future studies to further refine the mechanistic understanding of GRb_1_-mediated hepatoprotection.

In summary, in line with the traditional principle that prevention is better than cure, this study was designed within the holistic health framework. For the first time, real-life daily exposure of the population to pesticide residues was simulated to establish a model, and the preventive role of GRb_1_ in early liver injury was investigated. The official recognition of *P. ginseng* as a medicinal and edible resource has prompted increasing research interest in the health-promoting properties of its active constituents (Geng et al., 2024). As one of the major ginsenosides, GRb_1_ possesses well-documented antioxidant, anti-inflammatory (Tang et al., 2024), and immunomodulatory properties, and plays a critical role in cellular protection and repair. In this study, a modeled early liver injury was characterized by oxidative stress, nuclear envelope disruption, and DNA damage, even in the absence of overt clinical symptoms. Importantly, GRb_1_ effectively alleviated these adverse effects by activating the NHEJ DNA repair pathway, particularly at the stages of end recognition and ligation. In parallel, GRb_1_ restored nuclear membrane integrity and enhanced the expression of key nuclear envelope-associated proteins (Nesprin1, SUN1/2), thereby facilitating DNA repair and preserving genomic stability. The downregulation of Lamin A/C further indicated a reduction in cellular stress following GRb_1_ intervention. Notably, this study, for the first time within the holistic health framework, elucidated the protective mechanism of *P. ginseng* against liver injury induced by ubiquitous pesticide residues. Collectively, these findings provide experimental evidence for the cytoprotective role of GRb_1_ in liver injury and support the feasibility of ginsenoside-based early intervention strategies.

## CRediT authorship contribution statement

**Fei Gao:** Conceptualization, Methodology, Writing – original draft. **Haiyun Gao:** Data curation, Software. **Yun Lin:** Data curation, Software. **Longyan Chen:** Data curation, Software. **Zheng Qi:** Conceptualization, Writing – review & editing, Supervision, Funding acquisition. **Miao Yu:** Conceptualization, Writing – review & editing, Supervision, Funding acquisition.

## Declaration of competing interest

The authors declare that they have no known competing financial interests or personal relationships that could have appeared to influence the work reported in this paper.
