# Supplementary material for: Hepatoprotective effects of ginsenoside Rb1 from Panax ginseng on early liver injury via DNA repair activation and nuclear envelope stabilization
Source: Chin Herb Med. 2026 May 6;18(3):770–2. doi: 10.1016/j.chmed.2026.05.001 (PMC13389976; doi:10.1016/j.chmed.2026.05.001)
Supplement: Supplementary Data 1 [file mmc1.docx]

**Supplementary material**

**Text S1.** Materials and instruments.

Ginsenoside Rb1 (GRb1, 98.0% purity, HPLC, Lot No. 2231104005) was obtained from the Solarbio Science & Technology Co., Ltd. (Beijing, China). Acetamiprid (ACE) (97% purity, Lot No. RH591489) and glutathione (GSH, biotechnology grade, 99% purity, Lot No. R097302) were purchased from Rhawn Chemical Reagent Co., Ltd. (Shanghai, China). Detailed information on all chemicals was provided in Table S1, and the instruments used in the study were listed in Table S2.

**Text S2.** Animal handling and treatment.

Sixty male C57BL/6J mice [age: 4 weeks; body weight: 18−22 g; source: Beijing Vital River Laboratory Animal Technology Co., Ltd. SCXK (Jing) 2021-0006)]. The study protocol received ethical clearance from Harbin University of Commerce (No. HSDYY-2024-0032) in compliance with institutional animal care guidelines.

Male mice were selected based on the literature indicating that men have more stable hormonal levels than women. This suggests that hepatic metabolic enzymes such as cytochrome P450 (CYP450) are more stable and less affected by hormonal fluctuations, which reduces individual differences and improves data reproducibility (Zhang et al., 2024). To reflect experimental stability and ensure reliable evaluation of hepatic injury, male mice were used [e. g. pollutant-induced liver injury (Li et al., 2025), liver fibrosis and inflammatory response] (Wu et al., 2024).

Mice were randomly divided into six groups (*n* = 10) as follows: Control, model, GRb_1_ low-dose (GRb_1_-L, 12.5 mg/kg), medium-dose (GRb_1_-M, 25 mg/kg), high-dose (GRb_1_-H, 50 mg/kg), and GSH (50 mg/kg). Except for the control group (administered olive oil), all groups administered 5 mg/kg of ACE dissolved in olive oil by gavage once daily for 28 d. This dose reflect residue levels found in agricultural products and model environmentally relevant exposure.

Based on the correction factors including human variations of dose-responses (10 ×), the interspecies difference in toxicodynamics (human *vs* mice) (3 ×), and the interspecies difference in toxicokinetics (human *vs* mice) (81 ×), an equivalent murine dose of lowest tolerance level (0.01 ppm) was 24.30 mg/kg (Yang et al., 2025). The residual concentration of ACE in surface water is 2−410 μg/L (Eswaran, Bin Mamat, & Vasimalai, 2023) (the equivalent murine dose was calculated to 0.52 mg/kg corresponding to 2 μg/L). We thus set the exposure dose of 5 mg/kg as one-fifth of the minimum tolerated dose. Detailed grouping information was presented in Table S2.

**Text S3.** Preparation of hematoxylin and eosin (HE) stained sections.

Three samples each of liver, spleen, kidney, and lung tissues were randomly selected from each group, washed with phosphate-buffered saline (PBS), and fixed in 10% formalin, dehydrated through a graded ethanol series (75%, 85%, 95%, and 100%), embedded in paraffin, and sectioned at 3 μm thickness. The paraffin sections were dewaxed and stained with HE, and were observed under an inverted microscope for morphological evaluation. Sections of spleen, kidney, and lung were processed using the same procedure.

**Text S4.** Biochemical indicators analyses.

Serum levels of alanine aminotransferase (ALT), and aspartate aminotransferase (AST) were measured using an automated biochemical analyzer (Hitachi High Technologies Corporation, Japan). Levels of superoxide dismutase (SOD), catalase (CAT), and malondialdehyde (MDA) were measured using commercial kits according to the manufacturer’s instructions. Hepatic levels of 8-hydroxy-2′-deoxyguanosine (8-OHdG) were determined by the specific enzyme-linked immunosorbent assays. Results were presented in Table S3.

**Text S5.** mRNA-sequencing and analysis.

Total RNA was extracted from liver tissues and analyzed using a 2100 Expert Bioanalyzer. RNA sequencing was performed on the Illumina HiSeq 2500 platform.

**Text S6.** RNA extraction and quantitative reverse transcription-PCR.

Total RNA was reversed transcribed into cDNA using a commercial cDNA synthesis kit, following the manufacturer’s instructions. Quantitative real-time PCR was performed using TB Green^TM^ Premix Ex Taq^TM^ kit and a StepOnePlus^TM^ Real-Time PCR System. GAPDH was used as the internal control. The sequences of primers used were provided in Table S4.

**Text S7.** Alpha mouse liver 12 (AML12) cell culture and passaging.

AML12 cells, derived from mouse hepatocytes, were purchased from Shenzhen Haodi Huatuo Biotechnology Co., Ltd. and cultured in DMEM/F12 medium supplemented with 10% FBS, 10 µg/mL insulin, 5.5 µg/mL transferrin, 5 ng/mL selenium, 50 ng/mL dexamethasone, and 1% penicillin-streptomycin. The cells were incubated in a humid atmosphere containing 5% CO_2_ at 37 °C. When the cells reached approximately 80%−85% confluence, they were digested with trypsin-EDTA solution for passaging.

**Text S8.** Cell viability assay.

To explore the effect of GRb_1_ on AML-12 cells, cells were treated with GRb_1_ at concentrations of 0, 25, 50, 100, 200, or 300 μmol/L for 24 h. Briefly, the MTT assay was employed to assess the cell viability of AML12 cells. Initially, a 96-well culture plate was prepared, with each well containing 2.5  ×  10^4^ cells in 100 µL of culture medium. After 24 h, except the control group, the other five groups were treated with ACE (4.5 mmol/L) for 48 h. The vehicle control of 0.5% DMSO (*v*/*v*) was regarded as the concentration point of 0, the control group was the cells without any chemical treatment. After 48 h, GRb_1_ was added to each well (12.5, 25, 50 μmol/L of drug volume per well), and the positive drug was the cells with GSH (50 μmol/L). After 24 h, 20 µL of 5 mg/mL MTT solution was added to each well, resulting in a final concentration of MTT of 0.5 mg/mL. The plate was then placed in an incubator for 4 h, followed by careful removal of the supernatant. Subsequently, 100 µL of DMSO was added to each well. The plate was placed in a microplate reader, shaken for 5–10 min to facilitate the dissolution of the formazan crystals, and the optical density was measured at a wavelength of 490 nm. Results were expressed as the mean ± SD of six independent experiments.

Cell viability (%) = (*A*_Experiment_ – *A*_Blank_)/(*A*_Control_ – *A*_Blank_) × 100

**Text S9.** Nuclei and cell membranes fluorescent staining.

AML12 cells were seeded in 6-well plates at a density of 5 × 10⁵ cells/well and exposed to ACE (4.5 mmol/L) for 48 h. Subsequently, the cells were treated with different concentrations of GRb_1_ (25, 50, and 100 μmol/L; 0 μmol/L as the control) for 24 h. After treatment, the culture medium was removed and the cells were washed with PBS. For cell membrane staining, 1 mL of DIO staining solution (5 μg/mL) was added to each well and incubated for 30 min at 37 °C. The cells were then washed with PBS. For nuclear staining, the cells were incubated with Hoechst 33342 (10 μg/mL) for 30 min, followed by two PBS washes.

**Text S10.** Western blotting.

The expression levels of Nesprin1, SUN1, SUN2, and Lamin A/C were evaluated by Western blotting analysis. Fresh liver tissue samples were homogenized on ice in RIPA lysis buffer supplemented with a protease inhibitor cocktail to prevent protein degradation. The lysates were incubated on ice for 10 min and then centrifuged at 12 000 × *g* for 15 min at 4 °C to remove debris. The supernatants were collected for further analysis. Protein concentrations were determined using the BCA protein assay kit, following the manufacturer’s instructions to ensure equal protein loading. Equal amounts of protein (25 μg) were separated by SDS-PAGE on 8% polyacrylamide gels and transferred onto 0.45 μm nitrocellulose membranes. After that, the membranes were incubated in 5% milk in Tris-buffered saline (TBS) for 2 h before incubated with primary antibody on a shaker at 4 °C overnight. After three washes with Tris-buffered saline/Tween 20 (TBST), membranes were incubated with horseradish peroxidase (HRP)-conjugated secondary antibodies for 2 h at room temperature. Protein bands were visualized using Meilunbio^®^ FG Super Sensitive ECL Luminescence Reagent according to the manufacturer’s instructions. The relative protein expression levels of target proteins were quantified using Image-J software. Data visualization was performed using Origin 2024 software (OriginLab Corporation, Northampton, USA).

| **Table S1.** Reagents and manufacturers. |  |
| --- | --- |
| Reagents | Manufacturer |
| Dulbecco’s modified eagle medium: nutrient mixture F-12 (DMEM/F12) medium (batch number: MA0214) | Dalian meilun biotechnology Co., Ltd. (Dalian, China) |
| Penicillin-streptomycin solution (batch number: MA0110) | Dalian meilun biotechnology Co., Ltd. (Dalian, China) |
| Phosphate-buffered saline (PBS, batch number: MA0015) | Dalian meilun biotechnology Co., Ltd. (Dalian, China) |
| Dexamethasone (batch number: J0628D) | Dalian meilun biotechnology Co., Ltd. (Dalian, China) |
| Dimethyl sulfoxide (DMSO, batch number: D6370T) | Beijing biotopped science & technology Co., Ltd. (Beijing, China) |
| 0.25% trypsin-ethylenediaminetetraacetic acid (trypsin-EDTA, batch number: C0201) | Shanghai beyotime biotechnology Co., Ltd. (Shanghai, China) |
| 3-(4,5-Dimethylthiazol-2-yl)-2,5-diphenyltetrazolium bromide (MTT, batch number: ST316) | Shanghai beyotime biotechnology Co., Ltd. (Shanghai, China) |
| Fetal bovine serum (FBS, batch number: 23110201) | Zhejiang tianhang biotechnology Co., Ltd. (Huzhou, China) |
| Recombinant human insulin (batch number: I8830) | Beijing solarbio science & technology Co., Ltd. (Beijing, China) |
| Transferrin (batch number: T8010) | Beijing solarbio science & technology Co., Ltd. (Beijing, China) |
| Sodium selenite (batch number: W620545) | Shanghai wanvi biotechnology Co., Ltd. (Shanghai, China) |
| Protein-free cryopreservation medium (batch number: MA0401) | Dalian meilun biotechnology Co., Ltd. (Dalian, China) |
| Magnesium chloride (MgCl_2_, batch number: R051634) | Shanghai rhawn chemical reagent Co., Ltd. (Shanghai, China) |
| Potassium chloride (KCl, batch number: R018665) | Shanghai rhawn chemical reagent Co., Ltd. (Shanghai, China) |
| 4-(2-Hydroxyethyl)-1-piperazineethanesulfonic acid (HEPES) buffer (pH 7.4, batch number: R138071) | Shanghai rhawn chemical reagent Co., Ltd. (Shanghai, China) |
| 1,4-dithiothreitol (DTT, batch number: R050177) | Shanghai rhawn chemical reagent Co., Ltd. (Shanghai, China) |
| 4-(2-hydroxyethyl)-1-piperazineethanesulfonic acid (HEPES) buffer (pH 7.8, batch number: 4070262) | Shanghai saint-bio biotechnology Co., Ltd. (Shanghai, China) |
| Sucrose (batch number: R015038) | Shanghai rhawn chemical reagent Co., Ltd. (Shanghai, China) |
| Adenosine triphosphate (ATP, batch number: R014358) | Shanghai rhawn chemical reagent Co., Ltd. (Shanghai, China) |
| Protease inhibitor cocktail (100×, batch number: PI0015) | Beijing leagene biotech Co., Ltd. (Beijing, China) |
| Hoechst 33342 stain solution (batch number: C0030) | Beijing solarbio science & technology Co., Ltd. (Beijing, China) |
| 3,3′-dioctadecyloxacarbocyanine perchlorate (DiO, batch number: D5840) | Beijing solarbio science & technology Co., Ltd. (Beijing, China) |
| Absolute ethyl alcohol (batch number: 20240402) | Tianjin tianli chemical reagent Co., Ltd. (Tianjin, China) |
| Olive oil (batch number: 20230309) | Chongqing cuntou technology development Co., Ltd. (Chongqing, China) |
| Saline (batch number: 20240414) | Harbin triple pharmaceutical Co., Ltd. (Harbin, China) |
| Sodium chloride (NaCl, batch number: 20240504) | Tianjin hengxing chemical reagent manufacturing Co., Ltd. (Tianjin, China) |
| Meilunbio^®^ FG super sensitive enhanced chemiluminescence (ECL) luminescence reagent (batch number: MA0186) | Dalian meilun biotechnology Co., Ltd. (Dalian, China) |
| Bicinchoninic acid (BCA) protein assay kit (batch number: P0012S) | Shanghai beyotime biotechnology Co., Ltd. (Shanghai, China) |
| Skim milk powder (batch number: BS102) | Beijing bioSharp life sciences Co., Ltd. (Beijing, China) |
| Radioimmunoprecipitation assay (RIPA) lysis buffer (batch number: MA0151) | Dalian meilun biotechnology Co., Ltd. (Dalian, China) |
| Phosphatase inhibitor cocktail I (100×, batch number: MB12707-1) | Dalian meilun biotechnology Co., Ltd. (Dalian, China) |
| Sodium dodecyl sulfate-polyacrylamide gel electrophoresis (SDS-PAGE) sample loading buffer (5×, batch number: P0015L) | Shanghai beyotime biotechnology Co., Ltd. (Shanghai, China) |
| Tetramethylethylenediamine (TEMED) substitute (batch number: ST728) | Shanghai beyotime biotechnology Co., Ltd. (Shanghai, China) |
| Ammonium persulfate (APS, batch number: 20200620) | Tianjin beilian fine chemicals development Co., Ltd. (Tianjin, China) |
| Polysorbate 20 (Tween-20, batch number: A11445) | Tianjin alpha biotechnology Co., Ltd. (Tianjin, China) |
| Colormixed protein marker 180 (10−180 kDa, batch number: RM19001) | Abclonal technology Co., Ltd. (Wuhan, China) |
| SUN1 rabbit polyclonal antibody (pAb) (batch number: A16024) | Abclonal technology Co., Ltd. (Wuhan, China) |
| SUN2 rabbit monoclonal antibody (mAb) (batch number: A19782) | Abclonal technology Co., Ltd. (Wuhan, China) |
| Nesprin1 rabbit monoclonal antibody (mAb) (batch number: A19781) | Abclonal technology Co., Ltd. (Wuhan, China) |
| Lamin A/C rabbit monoclonal antibody (mAb) (batch number: A19524) | Abclonal technology Co., Ltd. (Wuhan, China) |
| Phospho-Lamin A/C-S22 rabbit polyclonal antibody (pAb) (batch number: AP0777) | Abclonal technology Co., Ltd. (Wuhan, China) |
| Glyceraldehyde-3-phosphate dehydrogenase (GAPDH) rabbit monoclonal antibody (mAb) (batch number: A19056) | Abclonal technology Co., Ltd. (Wuhan, China) |
| Horseradish peroxidase (HRP)-conjugated goat anti-rabbit immunoglobulin G (IgG) (H+L) (batch number: AS014) | Abclonal technology Co., Ltd. (Wuhan, China) |
| 30% acrylamide-bisacrylamide (29:1, batch number: ST003) | Shanghai beyotime biotechnology Co., Ltd. (Shanghai, China) |
| 1 M tris-hydrochloric acid (Tris-HCl) (pH 8.8, batch number: ST788) | Shanghai beyotime biotechnology Co., Ltd. (Shanghai, China) |
| 1 M tris-hydrochloric acid (Tris-HCl) (pH 6.8, batch number: ST768) | Shanghai beyotime biotechnology Co., Ltd. (Shanghai, China) |
| TB Green^TM^ Premix Ex Taq^TM^ kit (batch number: RR420A) | Takara biomedical technology Co., Ltd. (Beijing, China) |
| 10% sodium dodecyl sulfate (SDS) (batch number: ST628) | Shanghai beyotime biotechnology Co., Ltd. (Shanghai, China) |
| Sodium dodecyl sulfate (SDS, batch number: A2574) | Tianjin alpha biotechnology Co., Ltd. (Tianjin, China) |
| Tris (hydroxymethyl) aminomethane (Tris, batch number: A2575) | Tianjin alpha biotechnology Co., Ltd. (Tianjin, China) |
| Glycine (batch number: A1793) | Tianjin alpha biotechnology Co., Ltd. (Tianjin, China) |
| Methanol (batch number: 20240222) | Tianjin fuyu fine chemical Co., Ltd. (Tianjin, China) |
| 8-hydroxydeoxyguanosine assay kit | Nanjing chengjian institute of bioengineering (Nanjing, China) |

**Table S2****.** Instruments and manufacturers.

| Instruments | Manufacturer |
| --- | --- |
| Incubator (MCO-175) | Panasonic corporation (Osaka, Japan) |
| Laboratory super-pure water apparatus (EPED-E2-30TJ) | Nanjing yipu yida technology development Co., Ltd., (Nanjing, China) |
| Inverted microscope (CKX41) | Olympus corporation (Tokyo, Japan) |
| Analytical balance (Adventurer) | Ohaus corporation, (Parsippany, USA) |
| Electric-heated thermostatic water bath (DK-8D) | Shanghai yiheng technology instrument Co., Ltd., (Shanghai, China) |
| pH meter (PB-10) | Sartorius AG, (Goettingen, Germany) |
| Medical super cleaning worktable (DL-CJ-1N) | Beijing donglian har instrument manufacture Co., Ltd., (Beijing, China) |
| Microplate reader (iMark) | Bio-rad laboratories (Hercules, USA) |
| Inverted fluorescent microscope (IX73) | Olympus corporation (Tokyo, Japan) |
| Brightfield research microscope (DM4B) | Leica microsystems (Wetzlar, Germany) |
| Ultra low temperature freezer (Innova U725) | New brunswick scientific (Edison, USA) |
| Electrophoresis instrument (DYCZ-24DN) | Beijing liuyi biotechnology Co., Ltd. (Beijing, China) |
| Vertical electrophoresis tank (DYCZ-24DH) | Beijing liuyi biotechnology Co., Ltd. (Beijing, China) |
| Oscillator (TS-1000) | Haimen qilinbeier instrument manufacturing Co., Ltd. (Haimen, China) |
| Centrifuge (Anke TDL-80-2C) | Shanghai anting scientific instrument factory (Shanghai, China) |
| Electric thermostatic water bath (DK-89-1) | Tianjin taisite instrument Co., Ltd. (Tianjin, China) |
| Imaging system (c500) | Azure biosystems, Inc. (Dublin, CA, USA) |
| High-speed refrigerated centrifuge (L-100XP) | Beckman coulter, Inc. (Brea, CA, USA) |
| StepOnePlus^TM^ real-time PCR system | Thermo fisher scientific (Waltham, MA, USA) |
| 7180 clinical analyzer | Hitachi high-tech corporation (Tokyo, Japan) |
| Illumina HiSeq 2500 platform | Illumina, Inc. (San Diego, CA, USA) |
| Agilent 2100 bioanalyzer | Agilent technologies, Inc. (Santa Clara, CA, USA) |

**Table S3.** Biochemical indicators and liver 8-OHdG levels after GRb_1_ treatment ($\bar{x}\pm s$, *n* = 10).

| Group | ALT (U/L) | AST (U/L) | SOD (U/mg) | CAT (U/mg) | MDA (nmol/mg) | Relative level of 8-OHdG |
| --- | --- | --- | --- | --- | --- | --- |
| Control | 39.3 ± 2.83^***^ | 72.7 ± 6.5^***^ | 170.16 ± 8.7^***^ | 34.63 ± 1.25^***^ | 2.28 ± 0.22^***^ | 1.0 ± 0.01^***^ |
| Model | 78.0 ± 3.4 | 224.4 ± 10.8 | 76.78 ± 7.7 | 15.85 ± 1.15 | 5.54 ± 0.19 | 1.7 ± 0.06 |
| GRb_1_-L | 68.9 ± 3.67 | 198.0 ± 9.6 | 92.46 ± 7.6 | 20.41 ± 1.43 | 4.68 ± 0.20 | 1.5 ± 0.04 |
| GRb_1_-M | 63.9 ± 3.35^***^ | 154.3 ± 10.6^***^ | 128.56 ± 7.8^***^ | 25.19 ± 1.46^***^ | 3.45 ± 0.16^***^ | 1.3 ± 0.04^***^ |
| GRb_1_-H | 43.9 ± 3.00^***^ | 93.2 ± 7.1^***^ | 156.60 ± 6.6^***^ | 29.19 ± 1.26^***^ | 2.58 ± 0.20^***^ | 1.1 ± 0.05^***^ |
| GSH | 42.9 ± 2.77^***^ | 88.5 ± 6.6^***^ | 154.31 ± 6.9^***^ | 27.76 ± 1.52^***^ | 2.38 ± 0.18^***^ | 1.0 ± 0.04^***^ |

*^***^P <* 0.001 *vs* model group.

**Table S4.** Sequence of primers for RT-qPCR.

| Genes | Forward primer sequence (5’–3’) | Reverse primer sequence (5’–3’) |
| --- | --- | --- |
| *XRCC6* | GCAGTCTACTCCTGCCTAGTGA | ACCTGGCTCATCAAACCGCTTC |
| *RAD50* | TGGTAGACTGCCAGCGAGAACT | TCGGATGTGCTCTTGATGGCGA |
| *PRKDC* | AAGGCAGAAGCCTGGACAAGTG | ATCCGCCAGTAGGTCAATGCTG |
| *POLM* | GTCCGTTGCTCAGAAAGGTACC | GGTTCGTAGTCCTTCCTGGTAC |
| *DNTT* | CAGACAAAAGCCTGAGGTTTACAC | TGGTGACCAAGGCATCTGGAAG |
| *FEN1* | ACCAAGAGGCTCGTGAAGGTCA | GCAGCATAGACTTTGCCAGCCT |
| *XRCC4* | GCACCGATGAAGAAAGTGGAGC | GCATTCGGTGTCTCCTCTTCCT |
| *NHEJ1* | GTGCTGTTGGTGATGGAAAGCC | GCTGCTTGATGCCTGAGTCTCT |
| *GAPDH* | CATCACTGCCACCCAGAAGACTG | ATGCCAGTGAGCTTCCCGTTCAG |

**Table S5.** Cell viability of AML12 cells after ACE exposure ($\bar{x}\pm s$, *n* = 6).

| Groups | Concentration (mmol/L) | Cell viability (%) |
| --- | --- | --- |
| Control | — | 100.0 ± 0.0 |
| ACE | 1.25 | 85.2 ± 4.2*^***^* |
|  | 2.50 | 78.0 ± 8.5*^***^* |
|  | 5.00 | 68.6 ± 7.1*^***^* |
|  | 7.50 | 57.6 ± 4.2*^***^* |
|  | 10.00 | 45.9 ± 3.0*^***^* |

*^***^P <* 0.001 *vs* control group.

**Table S****6.** Cell viability of AML12 cells under GRb_1_ treatment ($\bar{x}\pm s$, *n* = 6).

| Groups | Concentration (mmol/L) | Cell viability (%) |
| --- | --- | --- |
| Control | — | 100.0 ± 0.0^***^ |
| ACE | 4.5 | 68.5 ± 4.5 |
| GRb1 | 12.5 | 73.7 ± 1.8 |
|  | 25 | 79.8 ± 1.6^***^ |
|  | 50 | 87.2 ± 2.2^***^ |
| GSH | 50 | 88.4 ± 2.3^***^ |

*^***^P <* 0.001 *vs* model group.

**Table S7.** Expression of nuclear membrane protein intervened by GRb_1_ ($\bar{x}\pm s$, *n* = 3).

| Group | Nesprin1 | SUN1 | SUN2 | Lamin A/C |
| --- | --- | --- | --- | --- |
| Control | 1.0 ± 0.1*^***^* | 1.0 ± 0.06*^***^* | 1.0 ± 0.06*^***^* | 1.0 ± 0.1*^***^* |
| Model | 0.2 ± 0.06 | 0.3 ± 0.1 | 0.5 ± 0.06 | 4.7 ± 0.06 |
| GRb_1_-L | 0.1 ± 0.06 | 0.4 ± 0.06 | 0.5 ± 0.06 | 4.6 ± 0.06 |
| GRb_1_-M | 0.3 ± 0.06 | 0.6 ± 0.06*^**^* | 0.8 ± 0.06*^***^* | 4.4 ± 0.06*^*^* |
| GRb_1_-H | 0.8 ± 0.1*^***^* | 0.8 ± 0.06*^***^* | 0.9 ± 0.06*^***^* | 3.9 ± 0.1*^***^* |
| GSH | 0.9 ± 0.06*^***^* | 0.8 ± 0.06*^***^* | 0.9 ± 0.06*^***^* | 3.8 ± 0.1*^***^* |

*^*^P <* 0.05, *^**^P <* 0.01, *^***^P <* 0.001 *vs* model group.

**Table S8.** Gene expression difference of NHEJ pathway compared with model group ($\bar{x}\pm s$, *n* = 3).

| Group | *XRCC6* | *RAD50* | *PRKDC* | *POLM* | *DNTT* | *FEN1* | *XRCC4* | *NHEJ1* |
| --- | --- | --- | --- | --- | --- | --- | --- | --- |
| Control | 640.48 ± 5.14*^***^* | 710.30 ± 14.00*^***^* | 461.00 ± 16.52*^***^* | 1 151.67 ± 14.57*^***^* | 1 033.33 ± 11.06*^***^* | 92.67 ± 17.56*^***^* | 511.33 ± 38.50*^***^* | 291.33 ± 26.16*^***^* |
| Model | 274.93 ± 8.88 | 198.83 ± 2.86 | 40.73 ± 5.87 | 221.31 ± 7.85 | 82.45 ± 9.15 | 196.33 ± 7.23 | 1 223.38 ± 37.01 | 807.63 ± 22.25 |
| GRb_1_-L | 280.65 ± 5.96 | 200.21 ± 2.12 | 48.78 ± 4.46 | 224.34 ± 7.03 | 88.82 ± 4.00 | 192.67 ± 15.01 | 1 806.90 ± 64.39 | 863.79 ± 20.22 |
| GRb_1_-M | 366.03 ± 4.38*^***^* | 244.26 ± 6.13*^***^* | 110.37 ± 5.67*^***^* | 325.85 ± 13.32*^***^* | 120.41 ± 9.20 | 234.67 ± 24.03 | 2 464.88 ± 29.88*^***^* | 1 141.81 ± 37.68*^***^* |
| GRb_1_-H | 561.37 ± 15.78*^***^* | 371.13 ± 7.73*^***^* | 178.08 ± 6.88*^***^* | 573.46 ± 18.53*^***^* | 217.96 ± 27.89*^***^* | 571.00 ± 22.34*^***^* | 2 756.91 ± 43.97*^***^* | 1 198.21 ± 20.87*^***^* |
| GSH | 318.66 ± 4.42*^***^* | 282.97 ± 11.75*^***^* | 264.86 ± 7.26*^***^* | 631.56 ± 19.96*^***^* | 251.16 ± 19.58*^***^* | 589.00 ± 30.45*^***^* | 2 357.87 ± 60.55*^***^* | 996.95 ± 12.42*^***^* |

*^***^P <* 0.001 *vs* model group.

**
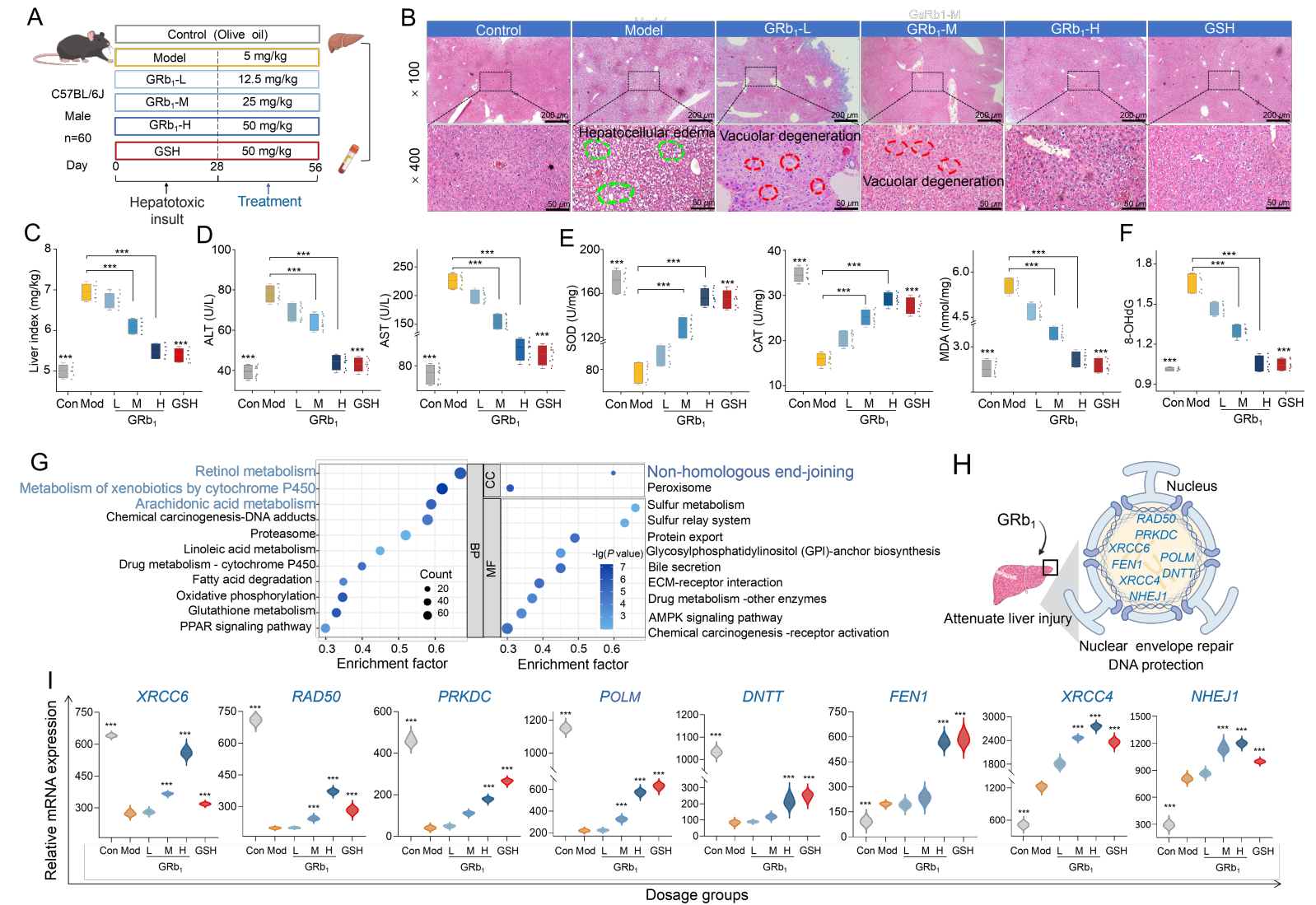
**

**Fig. S1.** GRb_1_ attenuates liver injury and improves hepatic function in mice. (A) Schematic illustration of the experimental design and GRb_1_ administration protocol (*n* = 10). (B) Representative hematoxylin and eosin (HE) staining images of liver tissue. Green squares indicate hepatocellular edema and red circles indicate vacuolar degeneration (scale bar = 100 μm at × 100, 50 μm at × 400). (C) Liver index analysis (*n* = 10). (D) Serum ALT, AST levels (*n* = 10). (E) Hepatic oxidative stress-related indicators, including SOD, CAT, and malondialdehyde (MDA) levels (*n* = 10). (F) Hepatic 8-OHdG levels reflecting DNA damage (*n* = 7). (G) KEGG pathway enrichment analysis of differentially expressed genes. (H) Schematic illustration of GRb_1_-mediated regulation of DNA repair through the NHEJ pathway. (I) Relative mRNA expression levels of key genes involved in the non-homologous end joining (NHEJ) DNA repair pathway. ^***^*P* < 0.001 *vs* Mod group. Con-Control; Mod-Model.


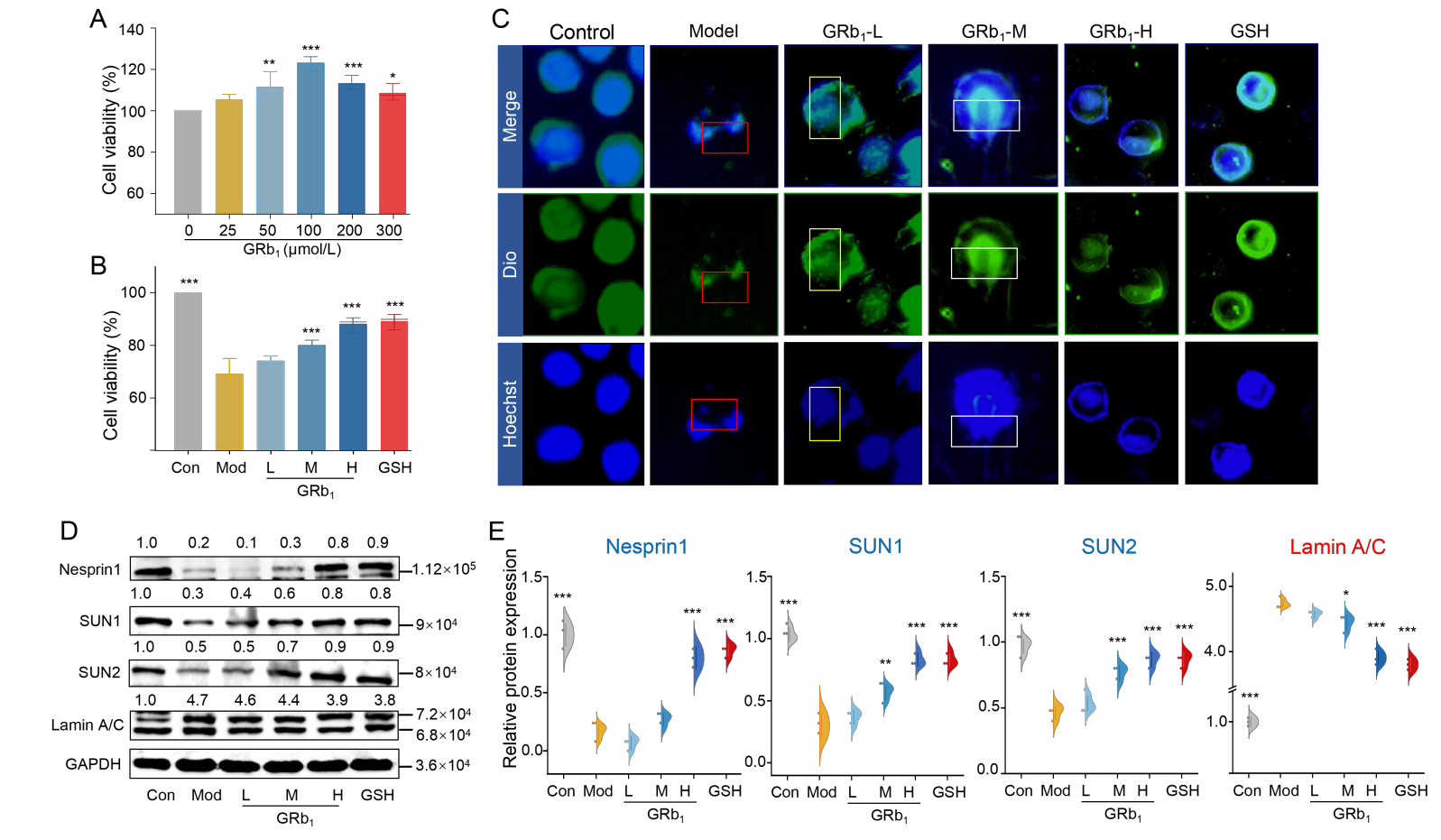


**Fig. S2.** GRb1 preserves nuclear integrity in AML12 cells under injury conditions. (A) Cell viability of AML12 cells treated with increasing concentrations of GRb1 (*n* = 6). (B) Cell viability of injured cells following GRb1 intervention (*n* = 6). (C) Representative fluorescence images showing nuclear (Hoechst, blue) and membrane (DiO, green) morphology under different treatments. (D) Representative immunoblots of nuclear envelope-associated proteins (Nesprin1, SUN1, SUN2, and Lamin A/C) (*n* = 3). (E) Quantification of protein expression levels. Data are presented as mean ± SD. *^*^P <* 0.05, ^**^*P <* 0.01, ^***^*P* < 0.001 *vs* Mod group. Con-Control; Mod-Model.


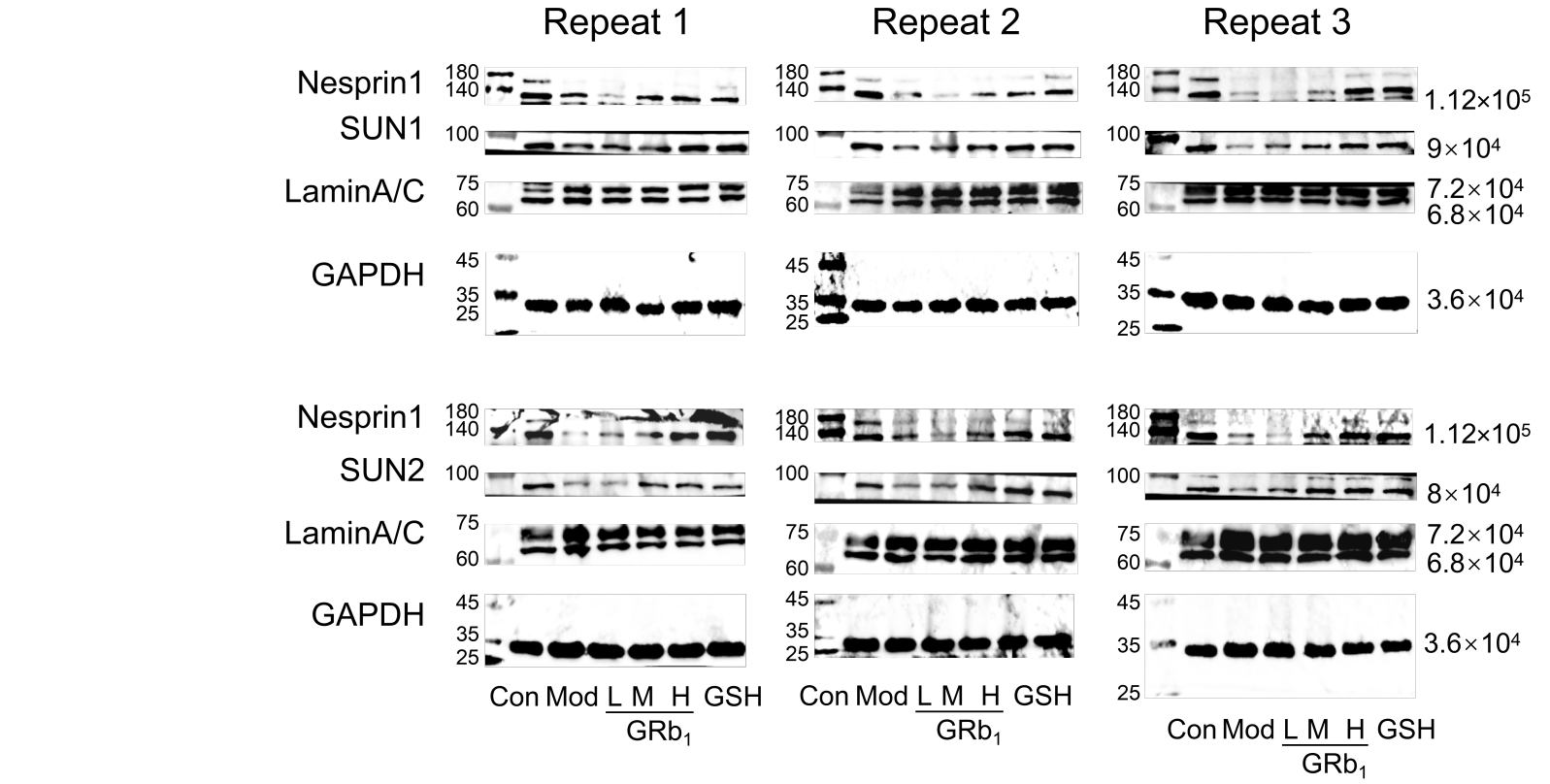


**Fig S3.** Protein expression levels of Nesprin1, SUN1, SUN2, and Lamin A/C intervened by GRb_1_ ($\bar{x}\pm s$, *n* = 3). Con-Control; Mod-Model.

**
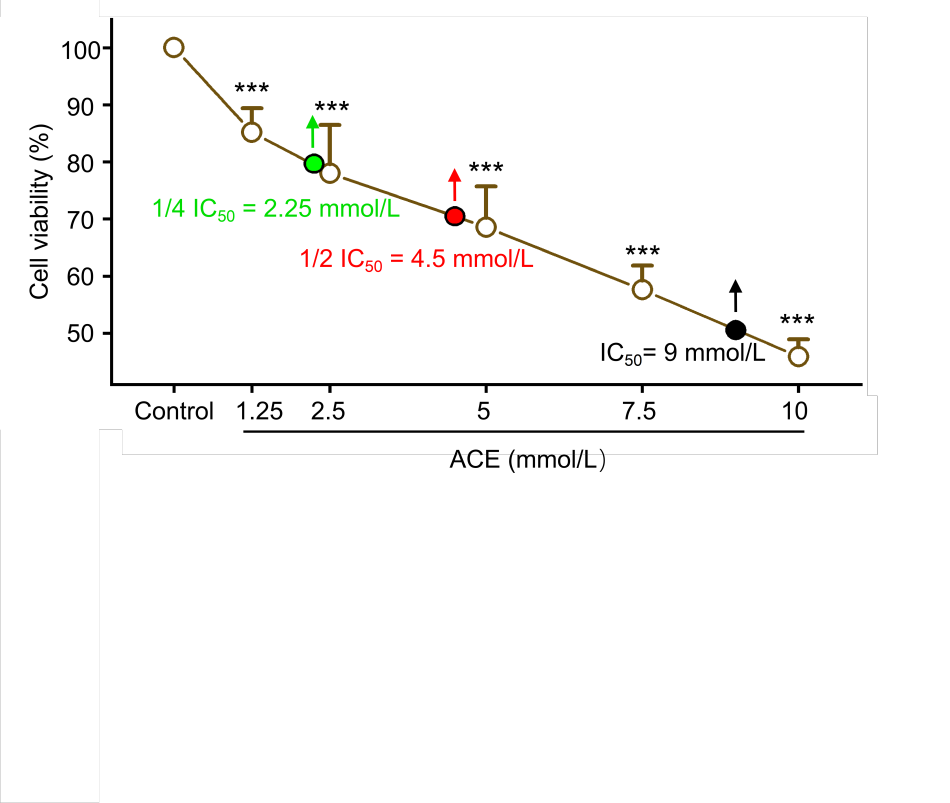
**

**Fig S4.** Cell viability of AML12 cells after ACE exposure ($\bar{x}\pm s$, *n* = 6)

**References**

Eswaran, S. G., Bin Mamat, M. H., & Vasimalai, N. (2023). Facile ultrasonication-assisted synthesis of Purpald-functionalized silver nanoparticles for the rapid spectrophotometric detection of acetamiprid pesticide in food and environmental samples. *Journal of Molecular Liquids*, *386*, 122425.

Li, M., Chen, S., Jiang, X. H., Ye, L. Z., Guo, Y. Z., Li, W. X., et al. (2025). Subchronic ozone exposure leads to multi-organ injuries with differential reversibility in male C57BL/6 J mice. *Journal of Hazardous Materials*, *492*, 138049.

Wu, X. N., Wang, M. Z., Zhang, N., Zhang, W., Dong, J., Ke, M. Y., et al. (2024). Sex-determining region Y gene promotes liver fibrosis and accounts for sexual dimorphism in its pathophysiology. *Journal of Hepatology*, *80*(6), 928–940.

Yang, W., Ma, K., Yin, S. P., Wang, W. H., An, H. H., Huang, Y. D., et al. (2025). Multiomic landscape of primary hypothyroidism induced by subchronic exposure to low-dose novel PFOS substitute OBS in human and murine models. *Environmental Science & Technology*, *59*(17), 8329–8344.

Zhang, M., Du, P. F., Xiao, Y. R., Liu, H., Wang, M. X., Zhang, Y. M., et al. (2024). Sex differences in CYP450-based sodium dehydroacetate metabolism and its metabolites in rats. *npj Science of Food*, *8*, 110.
